# Associations between UGT1A1, SLCO1B1, SLCO1B3, BLVRA and HMOX1 polymorphisms and susceptibility to neonatal severe hyperbilirubinemia in Chinese Han population

**DOI:** 10.1186/s12887-024-04537-0

**Published:** 2024-01-26

**Authors:** Juan Fan, Hua-Yun He, Huan-Huan Li, Pi-Liu Wu, Lei Tang, Bo-Yin Deng, Wen-Hui Dong, Jian-Hui Wang

**Affiliations:** 1 Department of Neonatology , Children’s Hospital of Chongqing Medical University, National Clinical Research Center for Child Health and Disorders, Ministry of Education Key Laboratory of Child Development and Disorders, Chongqing Key Laboratory of Child Rare Diseases in Infection and Immunity, Chongqing, China; 2 Department of Neonatology, Chongqing Jiulongpo People’s Hospital, Chongqing, China; 3 Department of Neonatology, Bishan Maternity & Child Hospital of Chongqing, Chongqing, China; 4 Department of Pediatrics, Affiliated Banan Hospital of Chongqing Medical University, Chongqing, China

**Keywords:** Single nucleotide polymorphism, UGT1A1, SLCO1B3, Neonatal hyperbilirubinemia

## Abstract

**Background:**

Severe neonatal hyperbilirubinemia could lead to kernicterus and neonatal death. This study aimed to analyze the association between single nucleotide polymorphisms in genes involved in bilirubin metabolism and the incidence of severe hyperbilirubinemia.

**Methods:**

A total of 144 neonates with severe hyperbilirubinemia and 50 neonates without or mild hyperbilirubinemia were enrolled in 3 institutions between 2019 and 2020. Twelve polymorphisms of 5 genes (UGT1A1, SLCO1B1, SLCO1B3, BLVRA, and HMOX1) were analyzed by PCR amplification of genomic DNA. Genotyping was performed using an improved multiplex ligation detection reaction technique based on ligase detection reaction.

**Results:**

The frequencies of the A allele in UGT1A1-rs4148323 and the C allele in SLCO1B3-rs2417940 in the severe hyperbilirubinemia group (30.2% and 90.6%, respectively) were significantly higher than those in the controls (30.2% vs.13.0%, 90.6% vs. 78.0%, respectively, both *p* < 0.05). Haplotype analysis showed the ACG haplotype of UGT1A1 were associated with an increased hyperbilirubinemia risk (OR 3.122, *p* = 0.001), whereas the GCG haplotype was related to a reduced risk (OR 0.523, *p* = 0.018).

**Conclusion:**

The frequencies of the A allele in rs4148323 and the C allele in rs2417940 are highly associated with the incidence of severe hyperbilirubinemia in Chinese Han neonates.

**Trial registration:**

Trial registration number:ChiCTR1800020424; Date of registration:2018-12-29.

## Introduction

Neonatal hyperbilirubinemia(NH) and resultant jaundice are commonly seen in 50–60% of newborns and, to a greater extent, in premature infants [1, 2]. Although most cases are benign and do not result in severe consequences, some infants will develop hazardous levels of bilirubin that directly threaten brain damage and may result in neuro-developmental abnormalities, such as hearing loss, athetosis, and intellectual deficit [2]. In pathological hyperbilirubinemia, increased production of bilirubin, deficiency in hepatic uptake, impaired conjugation of bilirubin, and/or increased enterohepatic circulation of bilirubin are observed [3]. Severe hyperbilirubinemia can be caused by maternofetal blood group isoimmunization (especially ABO hemolysis), G-6-PD deficiency, and severe infection [4]. Laboratory testing can predict this part of infants in advance and help guide active intervention to prevent exchange transfusion and reduce the risk of bilirubin encephalopathy. However, routine laboratory tests do not identify the etiology of severe hyperbilirubinemia in over 50% of infants [5, 6].

The American Academy of Pediatrics recommendations in 2004 identified East Asian ancestry, particularly mainland China, as a substantial risk factor for severe hyperbilirubinemia [7]. A positive family history can be a marker for shared genetic susceptibility; several studies [8, 9] have identified a previous sibling with a history of neonatal jaundice as an essential risk factor for neonatal hyperbilirubinemia. If an earlier sibling had a serum bilirubin > 15 mg/dL (257 µmol/L), the risk in subsequent siblings increased to 12.5-fold greater than controls [10].In addition, Ebbesen’s [11] research on bilirubin levels in identical and fraternal twins showed that after controlling for the factors known to regulate neonatal hyperbilirubinemia, genetics still has an important influence on neonatal bilirubin levels. Thus, genetic factors play an important role in the development of neonatal hyperbilirubinemia, especially genetic polymorphism of key enzymes involved in bilirubin metabolism. A growing body of evidence suggested that genetic variants in uridine diphosphate glucuronosyltransferase 1A1 (UGT1A1), solute carrier organic anion transporter family members 1B1 and 1B3 (SLCO1B1, SLCO1B3), glucose-6-phosphate dehydrogenase deficiency (G-6-PD), heme oxygenase 1 (HMOX1), and biliverdin reductase A (BLVRA) are closely associated with the incidence of severe hyperbilirubinemia.

However, the combined action of multiple genes on the occurrence of severe neonatal hyperbilirubinemia remains unclear. This study aimed to investigate the association between single nucleotide polymorphisms (SNPs) in genetic variants in bilirubin metabolism and the risk of severe hyperbilirubinemia.

## Materials and methods

### Patient characteristics

Between January 1, 2019, and June 30, 2020, 144 neonates admitted to three hospitals in southwest China with severe hyperbilirubinemia and meeting the following inclusion criteria were enrolled as the case group: (1) gestational age(GA) ≥ 35 weeks, within seven days of birth; (2) indirect bilirubin was the predominate component of serum bilirubin, accounting for more than 80% of the total bilirubin; (3) total bilirubin level met the exchange transfusion criteria in compliance with the guideline of management of hyperbilirubinemia endorsed by American Academy of Pediatrics in 2004 [7]. Meanwhile, a control group of 50 neonates was recruited, with GA ≥ 35 weeks and an age exceeding 7 days, who was in the absence of hyperbilirubinemia after brith or no phototherapy history. Additionally, their transcutaneous bilirubin levels monitored at community-based health facility after birth and blood bilirubin levels at admission were below the criteria requiring phototherapy [7]. Those with definite infection, multiple organ malformations, evident ABO or Rh incompatibility, and G-6-PD deficiency were excluded from this study. The experimental protocol was established, according to the ethical guidelines of the Helsinki Declaration and was approved by the Human Ethics Committee of the Children ‘s Hospital of Chongqing Medical University. Written informed consent was obtained from the parents or guardians.

### SNP selection

UGT1A1, SLCO1B1, SLCO1B3, HMOX1, and BLVRA genes were chosen for their crucial involvement in bilirubin metabolism. TagSNPs selected for the validation were based on the following criteria: (1) SNPs within 2 kb upstream and downstream of the gene area; (2) when multiple associated SNPs were in strong linkage disequilibrium (LD, r2 > 0.8), SNPs previously reported in the literature were prioritized. Twelve tagSNPs were chosen based on these parameters, including six from UGT1A1, two from SLCO1B1, two from SLCO1B3, one from BLVRA, and one from HMOX1(Table 1).

**Table 1:** Genotyping data for 12 tagSNPs genotyped from the candidate genes UGT1A1, SLCO1B1, SLCO1B3, BLVRA, and HMOX1

Gene (chromosome)	Reference SNP ID	Chromosome position	Region	MAF 1000G_CHB	MAF 1000G_EAS	Allele
UGT1A1(2)	rs4148323	234,669,144	exonic	0.192	0.138	A
	rs3771341	234,673,239	intronic	0.113	0.127	A
	rs34946978	234,676,872	exonic	0.024	0.011	T
	rs114982090	234,680,955	exonic	0.005	0.007	T
	rs35350960	234,669,619	exonic	0.007	0.014	A
	rs34993780	234,681,059	exonic	0.002	0.003	G
SLCO1B1(12)	rs4149056	21,331,549	exonic	0.127	0.123	C
	rs1564370	21,335,190	intronic	0.221	0.244	G
SLCO1B3(12)	rs2417940	21,017,875	intronic	0.173	0.195	T
	rs2117032	21,074,122	3’-flanking	0.421	0.461	C
BLVRA(7)	rs699512	43,810,764	exonic	0.310	0.317	G
HMOX1(22)	rs2071747	35,777,185	exonic	0.055	0.054	C

MAF: minor allele frequency, 1000G_CHB: Han Chinese in Beijing populations from the 1000 Genomes Project, 1000G_EAS: East Asian populations from the 1000 Genomes Project

### DNA isolation

Venous blood samples (2 to 3 ml) were collected and transferred to EDTA-anticoagulated tubes(Suzhou Bidi Medical Devices Co., Ltd.; Suzhou, China). The genomic DNA was isolated from EDTA-anticoagulated blood samples using the QIAamp DNA Kit (Qiagen, Hilden, Germany) and stored at − 80℃ for the subsequent experiments.

### SNP genotyping [12]

The genotyping of 12 SNP loci in one ligation reaction was done in this work using an enhanced multiplex ligation detection reaction method. The 12 SNP sites weres amplified using a multiplex of PCR procedures. The PCR reaction in 10 µl contained 1x Takara GC-I buffer, 3.0 mM MgCl_2_, 0.3 mM dNTP mix, 1 U HotStar Taq polymerase(Qiagen Inc.), 1 µl of primer mixture and 1 µl of genomic DNA.

The PCR program for all reactions was 95℃ for 2 min; 11 cycles x (94℃ 20 s, 65℃-0.5℃/cycle 40 s, 72℃ 1 min 30 s); 24 cycles x (94℃ 20 s, 59℃ 30 s, 72℃1 min 30 s); and 72℃ for 2 min; holding at 4℃. Exonuclease I and shrimp alkaline phosphatase, which were digested at 37 °C for 1 h and 75 °C for 15 min, were used to purify 10 µl of PCR products. The labeling oligo mixture, probe mixture, 2 µl of ligation buffer, 80 U of Taq DNA Ligase (NEB), and 5 µl of the purified PCR product mixture are all included in the ligation reaction, which is contained in 10 µl.

The ligation cycling program was 95℃ for 2 min; 38 cycles x (94℃ 1 min, 56℃ 4 min); holding at 4℃. A 0.5 µl of ligation product was loaded in an ABI3730XL, and the raw data were analyzed by GeneMapper 4.1.All of the primers, probes and labeling oligos were designed by and ordered from Genesky Biotechnologies Inc (Shanghai, China).

### Statistical analysis

Statistical analysis was performed by SPSS (version 19.0; SPSS, Inc., Chicago, IL, USA). Quantitative data were expressed as the mean ± SD and qualitative data as a percentage. Allelic frequencies were calculated by the gene-counting method. An unpaired Student’s t-test was utilized to compare the two groups. The Hardy-Weinberg test was used to evaluate the hereditary equilibrium. Linkage disequilibrium (LD) analyses were conducted using HaploView version 4.2 (Broad Institute, Cambridge, MA, United States) in Han Chinese in Beijing (CHB) populations from the 1000 Genomes Project phase 3 genotype data [13]. The chi-square test or Fisher’s precision probability test was used to assess the frequencies of alleles and genotypes in the two groups under three distinct genetic models: dominant, recessive, and additive. We used multivariable logistic regression adjusting for GA and age. The genotype relative risk was calculated using odds ratios (ORs) and 95% confidence intervals (CIs). Logistic regression analysis was used to calculate the significance of differences in genotype and allele frequencies and investigate the association of tested SNPs with hyperbilirubinemia risk. *P* < 0.05 was considered to be statistically significant.

## Results

### Clinical and demographic characteristics

A total of 194 newborns, including 144 neonates in the case group and 50 neonates in the control group, had blood samples obtained for DNA extraction and genotyping. All enrolled neonates were from the Chinese Han population,and did not receive any medications that may affect bilirubin metabolism during the perinatal period, such as phenobarbital. The demographic characteristics of the two groups are shown in Table 2. GA, birth weight, sex, and feeding patterns did not significantly differ between the groups. A significant difference in TSB and days of blood sampling was noted between the two groups because of the study grouping design.

**Table 2:** Clinical and demographic characteristics of neonates in Case and Control Groups

		Case Group (*N* = 144)	Control Group (*N* = 50)	*P* value
Gestational age (weeks)		38.4 ± 1.2	38.6 ± 1.4	0.519
Birth weight (g)		3117 ± 465	3204 ± 433	0.245
Gender (male/female)		69/75	28/22	0.325
feeding patterns	breast feeding	47	16	0.464
	artificial feeding	28	13	
	mixed feeding	69	21	
Days of blood sampling (days)		3.7 ± 1.8	16.5 ± 9.1	0.000
TSB(mg/dl)		20.8 ± 4.0	5.4 ± 3.7	0.000

TSB: Total serum bilirubin

### Hardy-Weinberg equilibrium test

Tests were performed for twelve loci between the groups, and the *P* values were > 0.05, indicating all loci were in Hardy-Weinberg equilibrium (Table 3).

**Table 3:** Hardy-Weinberg equilibrium in Case and Control Groups

Gene	Reference SNP ID	Case Group HWE-p	Control Group HWE-p
UGT1A1	rs4148323	0.766	0.818
	rs3771341	0.276	0.651
	rs34946978	1.000	1.000
	rs114982090	1.000	1.000
	rs35350960	1.000	1.000
	rs34993780	1.000	1.000
SLCO1B1	rs4149056	1.000	1.000
	rs1564370	1.000	0.452
SLCO1B3	rs2417940	1.000	0.506
	rs2117032	0.532	0.263
BLVRA	rs699512	0.318	0.617
HMOX1	rs2071747	1.000	1.000

HWE-p: *p* value for Hardy-Weinberg equilibrium

### Distribution of genotype, allele, and genetic susceptibility to hyperbilirubinemia of gene polymorphisms

Table 4 displays the allelic and genotypic frequencies of UGT1A1, SLCO1B1, SLCO1B3, BLVRA, and HMOX1 variants between the case and control groups. Significant differences in genotype and allele distributions in UGT1A1-rs4148323 and SLCO1B3-rs2417940 were noted between the two groups. Compared to the control group, the case group had higher frequencies of GA heterozygotes and AA mutant homozygotes at the rs4148323 locus (GA, 38.2% vs. 26.0%, *P* = 0.036 and AA, 11.1% vs. 0.0%, *P* = 0.003, respectively). Furthermore, the frequency of CC homozygotes was higher in the case group than the control group at the rs2417940 locus(81.3% vs. 58.0%, *P* = 0.002). Allele frequencies analysis revealed that the rs4148323 A allele in UGT1A1 gene and the allele C of SLCO1B3-rs2417940 were associated with hyperbilirubinemia (OR 2.897, *p* = 0.001 and OR 2.726,*P* = 0.001, respectively). However, there were no statistical differences in the genotype and allele distributions in UGT1A1-rs3771341, -rs34946978, -rs114982090, -rs114982090, SLCO1B1-rs4149056, -rs1564370, SLCO1B3-rs2117032, BLVRA-rs699512 and HMOX1-rs2071747 between the two groups.

**Table 4:** Genotypes and allele frequencies of SNPs in the UGT1A1, SLCO1B1, SLCO1B3, BLVRA, and HMOX1 genes

Genotypes/Alleles	Case group (*n* = 144) n (%)	Control group (*n* = 50) n (%)	*P* value	OR (95%CI)
UGT1A1-rs4148323				
GG	73 (50.7)	37 (74.0)		1
GA	55 (38.2)	13 (26.0)	0.036	2.144 (1.041–4.416)
AA	16 (11.1)	0 (0)	0.003^*^	0.820 (0.744–0.904)
A allele	87 (30.2)	13 (13.0)		
G allele	201 (69.8)	87 (87.0)	0.001	2.897 (1.535–5.465)
UGT1A1-rs3771341				
GG	134 (93.1)	42 (84.0)		1
GA	9 (6.2)	7 (14.0)	0.129^*^	0.403 (0.141–1.148)
AA	1 (0.7)	1 (2.0)	0.426^*^	0.313 (0.019–5.120)
A allele	11 (3.8)	9 (9.0)		
G allele	277 (96.2)	91 (91.0)	0.063	0.402 (0.161-1.000)
UGT1A1-rs34946978				
CC	136 (94.4)	48 (96.0)		
CT	8 (5.6)	2 (4.0)	1.000^*^	1.412 (0.290–6.882)
C allele	280 (97.2)	98 (98.0)		
T allele	8 (2.8)	2 (2.0)	1.000^*^	0.714 (0.149–3.421)
UGT1A1-rs114982090				
CC	138 (95.8)	50 (100.0)		
CT	6 (4.2)	0 (0)	0.342^*^	0.958 (0.926–0.992)
C allele	282 (97.9)	100 (100.0)		
T allele	6 (2.1)	0 (0)	0.345^*^	0.979 (0.963–0.996)
SLCO1B1-rs4149056				
TT	118 (81.9)	41 (82.0)		1
CT	25 (17.4)	9 (18.0)	0.934	0.965 (0.416–2.237)
CC	1 (0.7)	0 (0)	1.000^*^	0.992 (0.975–1.008)
C allele	27 (9.4)	9 (9.0)		
T allele	261 (90.6)	91 (91.0)	0.911	1.046 (0.474–2.308)
SLCO1B1-rs1564370				
CC	89 (61.8)	34 (68.0)		1
CG	48 (33.3)	13 (26.0)	0.354	0.709 (0.342–1.470)
GG	7 (4.9)	3 (6.0)	1.000^*^	1.122 (0.274–4.591)
C allele	226 (78.5)	81 (81.0)		
G allele	62 (21.5)	19 (19.0)	0.669	0.855 (0.482–1.517)
SLCO1B3- rs2417940				
CC	117 (81.3)	29 (58.0)		1
CT	27 (18.7)	20 (40.0)	0.002	2.989 (1.474–6.060)
TT	0 (0)	1 (2.0)	0.204^*^	1.034 (0.968–1.106)
C allele	261 (90.6)	78 (78.0)		
T allele	27 (9.4)	22 (22.0)	0.001	2.726 (1.471–5.054)
SLCO1B3-rs2117032				
CC	23 (16.0)	8 (16.0)		1
CT	66 (45.8)	30 (60.0)	0.565	1.307 (0.524–3.256)
TT	55 (38.2)	12 (24.0)	0.367	0.627 (0.227–1.737)
C allele	112 (38.9)	46 (46.0)		
T allele	176 (61.1)	54 (54.0)	0.212	0.747 (0.472–1.182)
BLVRA- rs699512				
AA	73 (50.7)	27 (54.0)		1
GA	60 (41.7)	18 (36.0)	0.550	0.811 (0.408–1.613)
GG	11 (7.6)	5 (10.0)	0.766^*^	1.229 (0.391–3.864)
A allele	206 (71.5)	72 (72.0)		
G allele	82 (28.5)	28 (28.0)	0.928	0.977 (0.589–1.620)
HMOX1- rs2071747				
GC	13 (9.0)	2 (4.0)		
GG	131 (91.0)	48 (96.0)	0.362^*^	2.382 (0.518–10.944)
C allele	13 (4.5)	2 (2.0)		
G allele	275 (95.5)	98 (98.0)	0.372^*^	2.316 (0.514–10.448)

*: Fisher’s precision probability test

### Linkage disequilibrium correlations of UGT1A1, SLCO1B1 and SLCO1B3 haplotypes with hyperbilirubinemia susceptibility

**Fig. 1:**
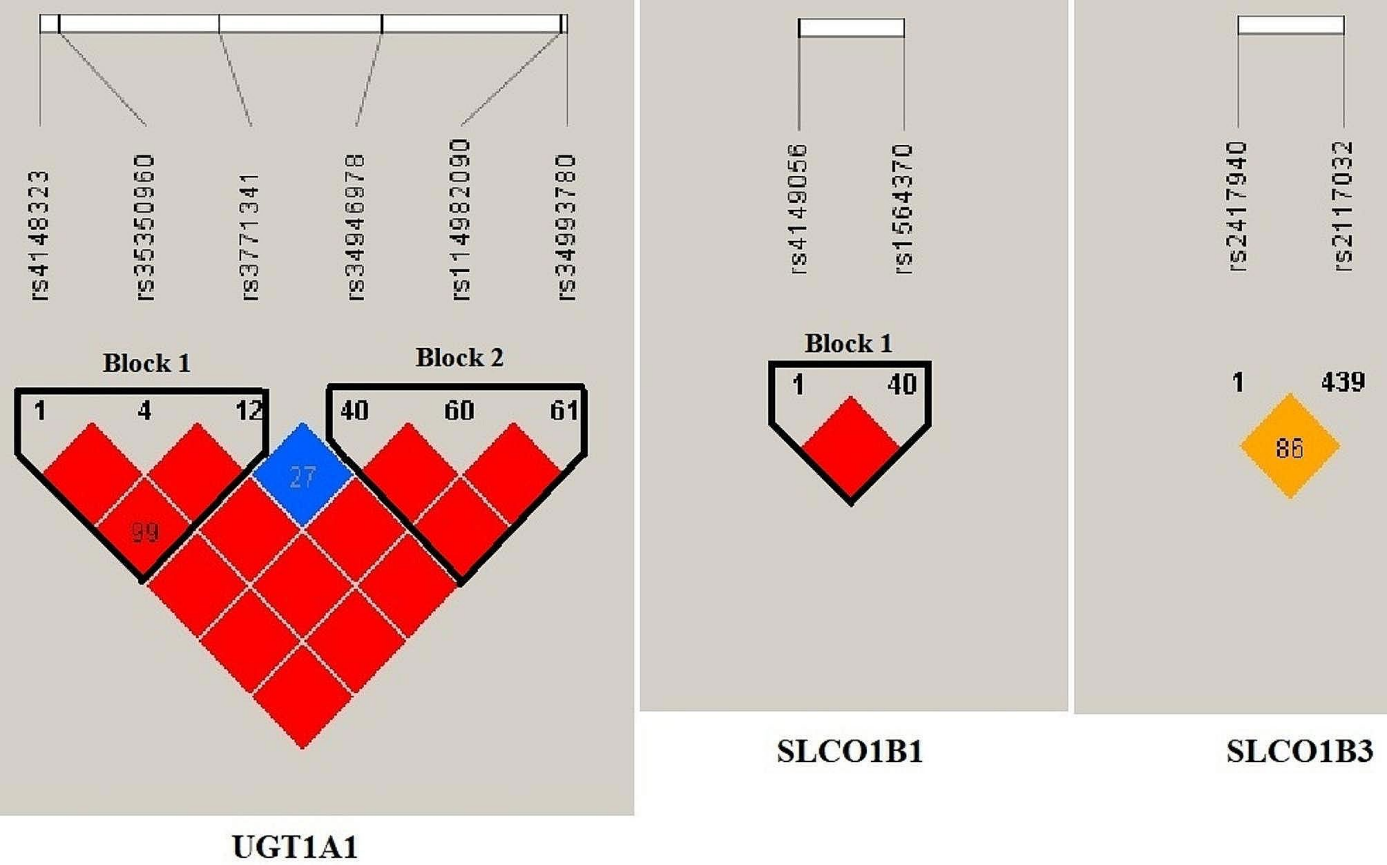
Linkage disequilibrium structures of SNPs of the candidate genes UGT1A1, SLCO1B1, and SLCO1B3

The linkage disequilibrium distributions of six UGT1A1 loci, two SLCO1B1 loci, and two SLCO1B3 loci are depicted in Fig. 1. Haplotypes were allocated based on linkage disequilibrium distribution, and relative distribution frequencies of haplotypes with in case and control groups were investigated. Two haplotypes of UGT1A1 were associated with hyperbilirubinemia susceptibility, of which ACG (OR 3.122, *p* = 0.001) was discovered to increase the risk of hyperbilirubinemia while GCG (OR 0.523, *p* = 0.018) reduced the risk. None of the haplotypes of SLCO1B1 and SLCO1B3 was found to be associated with hyperbilirubinemia (Table 5).

**Table 5:** Frequencies of haplotypes assigned by SNPs of UGT1A1, SLCO1B1, and SLCO1B3 genes in Case and Control Groups

Gene	SNPs constituted	Haplotypes	Frequencies		*P* value	OR (95%CI)
			Case group	Control group		
UGT1A1	rs4148323,rs35350960,rs3771341	GCA	0.034	0.080	0.114	0.414 (0.159–1.079)
		GCG	0.663	0.790	0.018	0.523 (0.305–0.898)
		ACA	0.004	0.010	0.450	0.345 (0.021–5.567)
		ACG	0.299	0.120	0.001	3.122 (1.624–6.003)
	rs34946978,rs114982090,rs34993780	CCT	0.951	0.980	0.343	0.399 (0.089–1.789)
		TCT	0.028	0.020	0.955	1.400 (0.292–6.706)
		CTT	0.021	/	0.345	/
SLCO1B1	rs4149056,rs1564370	CC	0.094	0.090	0.911	1.046 (0.474–1.373)
		TC	0.691	0.730	0.463	0.827 (0.498–1.373)
		TG	0.215	0.180	0.452	1.250 (0.698–2.238)

### Unconditional logistic analysis of SNPs and their susceptibility to hyperbilirubinemia

In the dominant model, UGT1A1-rs4148323 AA + GA genotypes were associated with an increased risk of hyperbilirubinemia (OR 2.768,*p* = 0.004). In the dominant model, the SLCO1B3-rs2417940 CC genotype was related to a higher risk of hyperbilirubinemia (OR 3.138, *p* = 0.001). There were statistical differences in the frequencies of the UGT1A1-rs4148323 AA genotype between the two groups by Fisher’s precision probability test. In the additive model, alleles of the SLCO1B3-rs2417940 minor C (OR 2.889, *p* = 0.003) were associated with an increased risk of hyperbilirubinemia. The frequencies of the remaining 10 SNPs did not differ between the two groups(Table 6).

**Table 6:** Comparison of allele and genotype distributions and frequencies in three genetic models between case and control groups

Gene	tagSNPs	Allele	Dominant model		Recessive model		Additive model		
			*P* value	OR (95%CI)	*P* value	OR (95%CI)	*P* value		OR (95%CI)
UGT1A1	rs4148323	A	0.004	2.768 (1.359–5.638)	0.013^*^	/	0.119	1.759 (0.860–3.598)	
	rs3771341	A	0.086	0.392 (0.145–1.057)	0.450^*^	0.343 (0.021–5.583)	0.131^*^	0.410 (0.144–1.165)	
	rs34946978	T	1.000^*^	1.412 (0.290–6.882)	/	/	1.000^*^	1.412 (0.290–6.882)	
	rs114982090	T	0.342^*^	0.958 (0.926–0.992)	/	/	0.342^*^	0.958 (0.926–0.992)	
SLCO1B1	rs4149056	C	0.993	1.004 (0.435–2.318)	1.000^*^	0.993 (0.980–1.007)	0.918	0.957 (0.413–2.218)	
	rs1564370	G	0.433	1.313 (0.663–2.599)	0.720^*^	0.800 (0.199–3.222)	0.336	1.423 (0.692–2.926)	
SLCO1B3	rs2417940	T	0.001	3.138 (1.558–6.321)	0.258^*^	1.020 (0.981–1.062)	0.003	2.889 (1.429–5.839)	
	rs2117032	C	0.069	1.957 (0.942–4.064)	0.996	1.002 (0.417–2.410)	0.084	0.564 (0.293–1.085)	
BLVRA	rs699512	G	0.687	0.876 (0.460–1.669)	0.563^*^	1.343 (0.443–4.076)	0.481	1.270 (0.653–2.471)	
HMOX1	rs2071747	C	0.362^*^	2.382 (0.518–10.944)	/	/	0.362^*^	2.382 (0.518–10.94)	

*: Fisher’s precision probability test

## Discussion

Hyperbilirubinemia, presenting as jaundice, is a ubiquitous and frequently benign condition in newborn babies, albeit a leading cause of hospitalization in the first week of life [14]. Although the exact etiology of hyperbilirubinemia remains unclear [15], the role of genetic factors in the pathogenesis of hyperbilirubinemia has received significant attention from neonatologists [16]. The current study aims to determine the roles of several genetic bilirubin metabolism modifiers in developing severe hyperbilirubinemia in Chinese Han newborns. Our research indicated striking relationships between the vulnerability to severe hyperbilirubinemia and the gene polymorphisms for UGT1A1 and SLCO1B3.

UGT1A1 is a member of the UGT1 family of microsomal membranes and plays an essential role in converting the toxic form of bilirubin to its nontoxic form [17]. The present study demonstrated that rs4148323 in the UGT1A1 gene is independently associated with total bilirubin levels. The frequency of the A allele in rs4148323 was associated with the incidence of severe hyperbilirubinemia. A prior study has reported that the A allele in rs4148323 is common in the East Asian population, with allele frequencies of 19.2% in Han Chinese and 19% in Korean populations [18], and 16.2% in the Japanese population as well [19], whereas it is monomorphic in the European populations (http://www.ncbi.nlm.nih.gov/snp), indicating that rs4148323 might be specifically associated with serum bilirubin levels in Asians. In the control group, the A allele frequency was 13%, similar to another study in China [20], and is much higher than in the severe hyperbilirubinemia group which had a frequency of 30.2%, similar to that observed in Japanese studies [21].

SLCO1B3, located at 12p12, is highly expressed in the basolateral hepatocyte membrane. It is an organic anion transporter gene coding an organic anion transporter polypeptide (OATP/1B3) that mediates the extrusion of bilirubin [22]. Genetic variants of SLCO1B3 contributed to idiopathic mild unconjugated hyperbilirubinemia [23]. The variant of rs2417940, located in intron 7 of SLCO1B3, was significantly associated with total serum bilirubin levels [18]. Our study demonstrated that the genotype CC and the frequency of allele C in the SLCO1B3-rs2417940 significantly correlated to the incidence of hyperbilirubinemia (81.3% vs. 58.0%, *P* = 0.002, OR 2.989 and 90.6% vs. 78.0%, *P* = 0.001, OR 2.726, respectively). Dai et al. [24] reported that smoking and rs2417940 polymorphism in SLCO1B3 on total bilirubin levels had a significant interaction, and rs2417940 had a much stronger effect on serum bilirubin levels in nonsmokers than in smokers. The frequency of allele T in the control group with mild or no hyperbilirubinemia was 22.0%, similar to another report in China (18.3%) [25].

We acknowledge that this study had several limitations. In this article, only twelve loci of genes that may affect bilirubin metabolism were assessed, including UGT1A1, SLCO1B1, SLCO1B3, HMOX1, and BLVRA. It is uncertain whether the polymorphism of other SNPs in the above genes or other genes would affect the serum bilirubin level. However, to our best knowledge, the current analysis was cost-effective, containing all relevant variations in these genes described in the Asian population. Another obvious limitation is that we could not examine the effect of medications on the metabolism of bilirubin levels or the activity of the target genes. However, medications are rarely prescribed for most neonates with hyperbilirubinemia in clinical settings.

## Conclusion

This study demonstrated the influence of genetic polymorphisms of several hyperbilirubinemia-related genes, illuminating the complicated nature of this condition. However, severe neonatal hyperbilirubinemia is a multifactorial issue. Future studies focusing on the interactions of the bilirubin metabolism gene, other genes, and nongenetic factors will provide a more holistic insight into the pathogenesis of neonatal hyperbilirubinemia.

## Data Availability

Full data set and other materials on this study can be obtained from the corresponding author on reasonable request.

## Abbreviations

UGT1A1: Uridine diphosphate glucuronosyltransferase 1A1
SLCO1B1: Solute carrier organic anion transporter family members 1B1
SLCO1B3: Solute carrier organic anion transporter family members 1B3
HMOX1: Heme oxygenase 1
BLVRA: Biliverdin reductase A
SNPs: Single nucleotide polymorphisms
GA: Gestational age
LD: Linkage disequilibrium
MAF: Minor allele frequency
1000G_CHB: Han Chinese in Beijing populations from the 1000 Genomes Project
1000G_EAS: East Asian populations from the 1000 Genomes Project
TSB: Total serum bilirubin
HWE-p: *p* value for Hardy-Weinberg equilibrium
